# The role of sense of presence in expressing cognitive abilities in a virtual reality task: an initial validation study

**DOI:** 10.1038/s41598-023-40510-0

**Published:** 2023-08-17

**Authors:** Tommaso Palombi, Federica Galli, Francesco Giancamilli, Monica D’Amico, Fabio Alivernini, Luigi Gallo, Pietro Neroni, Marco Predazzi, Giuseppe De Pietro, Fabio Lucidi, Antonio Giordano, Andrea Chirico

**Affiliations:** 1 Department of Psychology of Developmental and Socialization Processes, “Sapienza” University of Rome, Via Dei Marsi, 78, 00185 Rome, Italy; 2 Department of Movement, Human and Health Sciences, University of Rome, Foro Italico, Rome, Italy; 3 Department of Clinical Psychology, “Sapienza” University of Rome, Rome, Italy; 4 Institute for High-Performance Computing and Networking, National Research Council, Naples, Italy; 5 Fondazione Il Melo Onlus, Gallarate, Italy; 6 Sbarro Institute for Cancer Research and Molecular Medicine, Center for Biotechnology, College of Science and Technology, Temple University, Philadelphia, PA USA; 7 Department of Medical Biotechnologies, University of Siena, Siena, Italy

**Keywords:** Psychology, Health care

## Abstract

There is a raised interest in literature to use Virtual Reality (VR) technology as an assessment tool for cognitive domains. One of the essential advantages of transforming tests in an immersive virtual environment is the possibility of automatically calculating the test’s score, a time-consuming process under natural conditions. Although the characteristics of VR can deliver different degrees of immersion in a virtual environment, the sense of presence could jeopardize the evolution of these practices. The sense of presence results from a complex interaction between human, contextual factors, and the VR environment. The present study has two aims: firstly, it contributes to the validation of a virtual version of the naturalistic action test (i.e., virtual reality action test); second, it aims to evaluate the role of sense of presence as a critical booster of the expression of cognitive abilities during virtual reality tasks. The study relies on healthy adults tested in virtual and real conditions in a cross-over research design. The study’s results support the validity of the virtual reality action test. Furthermore, two structural equation models are tested to comprehend the role of sense of presence as a moderator in the relationship between cognitive abilities and virtual task performance.

## Introduction

In recent years, the use of virtual reality (VR) technology and immersive interface have attracted the interest of several scholars^1–3^. VR has been traditionally defined as “interactive, virtual image displays enhanced by special processing and by non-visual display modalities […] to convince users that they are immersed in a synthetic space”^4^. The primary purpose of this technology is to provide an authentic and immersive experience, replacing real stimuli with high realism that can be customized and integrated into an ecological task^5–7^. The simulated environment can be easily manipulated, facilitating experimental tasks that are difficult to implement in real-world settings. Other benefits of VR regard the possibility of participating in potentially dangerous tasks, such as moving in a complex environment or applying psychological treatment to problems arising from phobias^8^ in a controlled ecological setting^9^. Although VR technology has an interdisciplinary nature, in the last few years, it might appear that most developments in VR studies have focused on clinical aspects^5^. Nowadays, VR emerges as a promising valuable technology as an effective medium for administering different interventions in the healthcare contexts^10,11^. Moreover, VR is considered a valuable tool that could improve and automatize the processes of administering and scoring traditional performance-based assessments without jeopardizing ecological validity. Different research highlighted that the score calculated from VR devices reduces the number of errors that could distort the results^12^ and reduces the time required for the scoring^11^. The technological progress of VR has expanded the range of tools and types of research questions, adapting several standard performance-based tests to the virtual version. Different studies applied VR tools to assess executive functions^13^ and spatial abilities^5,14^, showing promising results. However, several performance-based tests are traditionally delivered in real-life settings (e.g., executive functions performance test—EFPT^15,16^; the multiple errands test—MET^17–19^; radial arm maze task—RAM^20^) this practice was often revealed as time-consuming and not always feasible^21^.

The extent to which an individual can manage daily tasks and perform everyday life activities is heavily influenced by their cognition. To be able to meet the demands of daily life, a person must possess the ability to remember, concentrate, plan, and reason. The link between cognition and functionality has been demonstrated not only in individuals with cognitive impairment^22^ or mental health disorders but also in healthy individuals^23^. As a result, the scientific literature showed that it is important to take cognition into consideration when attempting to comprehend daily patterns or support functioning. Typically, clinical or laboratory settings are used to evaluate memory functions, processing speed, and other cognitive abilities rather than natural environments^24^. Several studies highlighted that traditional neuropsychological assessments appear to have little relevance to the real-life difficulties individuals experience in their daily life^24,25^. This conventional approach may thus impact the ecological validity of neuropsychological test results^26^.

Performance-based tests try to overcome the conventional approach, specifically everyday life assessment involves the evaluation of several cognitive domains, often using multiple tasks related to the everyday activities of an individual (e.g., preparing a meal). Several studies showed that the strategy used to complete a task is different for different tasks involving mainly processing speed or memory^24^. Memory and processing speed are the most prevalent cognitive domains assessed, which may be attributed to their relevance both for various clinical^27,28^ and healthy populations^29,30^. Memory is mainly related to the ability to maintain, update, and manipulate information in an active state, recalling scripts needed to complete a multi-step action. On the other side, processing speed is involved in the execution of fine behaviors and gestures, and the so-called “perceptual speed”^31^. Considering performance base tests of everyday life activities, memory and processing speed could be related to different performance patterns, the first mainly linked with the steps to accomplish (e.g., preparing a meal), the latter related to motor behavior accuracy/errors^32,33^. There is a significant body of scientific literature that supports the separation of memory and processing speed in experimental designs^34–37^, overall the literature supports the hypothesis of the “independent factor model”, supporting a separate evaluation of memory and processing speed in experimental designs.

One of the most widely used performance-based tests is the Naturalistic Action Test (NAT), a valid and reliable test that measures the functional abilities of everyday activities in order to assess any inefficiencies (e.g., mis-reaching an object^38–40^). The NAT is sensitive to mild functional changes associated with cognitive aging^41,42^ and includes tasks of increasing complexity, such as lunch preparation^38,40^. The NAT scoring system evaluates both the steps performed for the task and the errors identified in the action. Recently, Giovannetti et al. developed a non-immersive digitalized version of NAT designed with a touchscreen interface in which the scoring was automatically calculated by the software without the need for human coding^43^. In a pilot study conducted by Giovannetti et al. the results showed a high sensitivity of the test in terms of predicting significant group differences in mild functional difficulties^43^. Non-immersive systems include the development of a 2D virtual environment projected on a desktop to reproduce images of the world, however, non-immersive systems lack realism, and the interactions between the subject and the digital objects are completely different from the real motor behavior^5^. In particular, immersion involves stimulating senses, interactions, and reality's similarity to the stimuli used in virtual environments. This feature can depend on the properties of the technological system used to isolate the user from reality^5^. Although the characteristics of VR technology can deliver different degrees of immersion in a virtual environment, the sense of presence experienced by participants is the result of a complex interaction between human factors, contextual factors, and the VE^44,45^. The sense of presence plays an important role in the VR experience, and it has been noted that it is often complicated to find a direct influence on performance in VR tasks^44,46^. The scientific literature makes no definitive claims about the relationship between presence and performance^47–51^. Several studies have reported a positive relationship between presence and performance in a variety of virtual environments and tasks^52–54^. In a study conducted by Cooper and colleagues^52^, participants were instructed to perform a wheel change simulation task in VR, those who reported experiencing a higher level of sense of presence performed better (task completion time). However, other experimental studies have been unsuccessful in finding a positive relationship between sense of presence and performance, showing a weak or null relationship^55–58^. In a recent study conducted by Voinescu et al., the authors reported a null effect of sense of presence on performance in VR task^55^. The findings related to the relationship between sense of presence and performance are frequently inconsistent^59,60^, suggesting that this relationship depends on the nature of the performance and its relationship with several individual factors.

Considering the above, evaluating the role of sense of presence in the analysis of performances in virtual performance-based tests predicted by cognitive tests becomes important. Since having the feeling of being present in a Virtual Environment is not related to the device per se, but it is a complex interaction between individual and contextual factors, different individuals could feel different degrees of sense of presence with the same experimental condition. However, feeling in a VE means also expressing own behavior during the natural (ecological) performance of activities, and this is especially important when it comes to using VR to assess everyday life activities (e.g., preparing a meal), where the ecological condition is crucial^61^. Whether the sense of presence reported null or weak direct effects on scores of performance-based tasks, it stands to reason that it could act as a moderator between individual abilities and their expression in terms of performance in a virtual environment. In line with the above, the present study aimed to test a virtual immersive version of the NAT (i.e., Virtual Reality Action Test, VRAT), following the same protocol implemented by Chirico and colleagues (2020). Using Head-Mounted Display (HMD) devices, we administered the VRAT to a sample of healthy adults. Participants have been asked to perform the same task (i.e., breakfast) in a cross-over trial in both conditions: virtual vs. real. Moreover, we administered a cognitive battery test to assess the participants’ cognitive function. Based on previous research^41^, we hypothesized that cognitive tests could predict performance in VRAT. Moreover, we expected that the VR experienced by participants (i.e., sense of presence) could moderate the relationship between cognitive tests and VRAT performance. Finally, in line with the literature^43^, we expected significant correlations between performances in both virtual and real tasks and cognitive tests.

## Materials and method

### Participants

The study sample was composed of 16 adults. Participants were recruited between December 2019 and March 2020. Exclusion criteria for both groups were as follows: non-Italian speaker, current or past neurological disorder or major medical illness (e.g., dementia, traumatic brain injury, schizophrenia, epilepsy, active nausea, vomiting), current psychiatric disorder (e.g., major depression), a severe sensory or motor deficit that would preclude interaction with devices, and history of previous motion sickness due to exposure to VR, TV or similar. Inclusion/exclusion criteria were evaluated via a self-report questionnaire at the time of recruitment and a brief interview following informed consent obtained from all participants. The study was approved by the Ethical Committee (Department of Psychology of Developmental and Socialization Processes at “Sapienza”, University of Rome). All methods were carried out in accordance with relevant guidelines and regulations and in accordance with the Declaration of Helsinki.

### Procedure

All procedures were identical for all participants in a cross-over design and were completed in a single session. The procedure was completed in a single 2-h session. Written informed consent was obtained by the participant, which specified all the risks related to VR, specifically motion sickness. The participants were asked if they have ever experienced any symptoms of motion sickness in past virtual reality experiences or other visual devices (i.e., television, video games). Before each condition, the participants completed a 5-min training session with the VR system and with the real objects present on the table. In the virtual environment, when the virtual hand reaches an object, the object is highlighted to inform the user through visual feedback that it is selected and interactable. To interact with a virtual object in the VRAT, the user is instructed to press the trigger button once the object is highlighted/selected. To end the interaction, the user is instructed to release the trigger. Training included two mini-tasks regarding the manipulation of virtual and real objects. The mini-tasks were not related to the task performed during the trial. Once participants were familiar with the real and virtual environment, the experimenter gave instructions to perform the two versions of the task. Afterward, the experimenter would leave the laboratory in order to go to an adjacent control room to observe the experiment through a camera. The procedure was identical between the two conditions. Participants completed the test on the VRAT with controllers and its real version (order counterbalanced). At the end of the task, the presence questionnaire (only following the VRAT), cognitive tests, and other questionnaires were administered.

### Performance-based functional tests

The breakfast task was administered in a highly immersive virtual environment and in a real-world environment, following the administration of NAT^40^. The breakfast task requires participants to prepare a slice of toast with butter and jelly and a cup of coffee with milk and sugar while seated at a table containing a toaster, two knives, one spoon, butter in a butter dish, sugar in a bowl, a bottle of milk, mug filled with warm water, bread, instant coffee, jelly jar, and a napkin at the central workspace. The shape of the table and the spatial arrangement of objects followed the procedures reported in the NAT manual (NAT Manual; https://mrri.org). The breakfast task was administered in real and virtual environments. In both conditions, participants were instructed to complete the task as quickly as possible and without making errors. They were asked to make their movements as clear as possible and to tell the examiner when the task is finished. Performance in real conditions was video-recorded for scoring.

### Virtual reality action test (VRAT)

The VRAT is an immersive VR task that includes an everyday task (i.e., preparing breakfast) designed to maximize ecological validity by simulating real kitchen and household objects. The VRAT environment includes accurate 3D models, spatial audio, and automatic and real-time collection of movement data. The VR system included the HTC Vive head-mounted display that provided a fully immersive experience in a virtual environment and the controllers that provided tactile feedback through vibration to enable interaction with virtual objects in the VRAT. The VRAT system runs on an MSI Trident Gaming Desktop with 8 GB RAM and a GTX 1060 graphic card. The HTC Vive head-mounted display provides users with a fully immersive virtual environment. The system provides visual content through two OLED displays for a total resolution of 2160 × 1200 pixels with a 110-degree FoV and a frequency of 90 Hz. The equipment and software specifications were the same used in a recent case study conducted by Chirico and colleagues^11^.

During the VRAT, the participants were in a seated position in front of a virtual desk with virtual objects. The controllers were used to interact with the VR environment in which the user’s hand motions are directly mapped to the virtual hand movements. As soon as the virtual hand reaches an object, the object is highlighted to indicate that it is selectable and interactable. Users are instructed to press a trigger button to interact with virtual objects in VRAT and release the trigger to end the interaction. The first phase of the VRAT regarded a VR training session in order to familiarize participants with the virtual environment. The training included four mini-tasks that comprised elements of the breakfast task: (1) toast a slice of bread; (2) spread the jelly on toast; (3) add instant coffee to a cup; (4) add milk to a cup. The examiner controlled the presentation of each mini-task from a monitoring position and could correct errors in object identification or the performance of task steps. The participants were encouraged to ask any questions. After the VR training session, the participants completed a single test trial of the VRAT in which they have been asked to perform the breakfast task. Participants were instructed to complete the test trial in silence, as quickly as possible, without making errors, making their movements as clear as possible. At the end of the task, they were told to stop and declare the test over.

Although the VRAT includes the error monitoring module, performance quality, and accuracy on the real condition and virtual conditions were evaluated by three trained coders, who independently viewed and coded the recordings of the participants’ performances to ensure inter-rater agreement. The performance scores were composed by:

- Total errors: incorrect actions (commission), the failure to complete a step (omission), and off-task actions (additions).
- Micro-errors: inefficient but not overtly incorrect actions; this category of errors refers to subtle inefficient behaviors in performing the task (e.g., reaching an object not needed for the accomplishment of the step).
- Accomplishment score: an accomplishment point was assigned for each task step of the breakfast task completed without error (range = 0–16).

### Virtual reality measures

After the VRAT conditions, questionnaires about the experience were administered to the participants.

#### Presence questionnaire

The participants were asked to complete the Italian version of the Presence Questionnaire^62^. The questionnaire, in its original form, comprises 24 items that explore different aspects of the VR experience, rated on a 7-point Likert scale. The factors are distributed as follows: 7 items on Realism; 4 items on the Possibility to act; 3 items on the Quality of the interface; 3 items on the Possibility to examine; 2 items on Self-evaluation of performance. In this study, we used the following factors: Realism, the Possibility to act, and the possibility to examine. Reliability was higher than 0.88 for each factor.

#### Cybersickness symptoms

The Italian version of the VR Cybersickness Symptoms^63^ was proposed to the participants to evaluate two types of side effects caused by exposure to VR: visual effects, such as tired eyes, aching eyes, eyestrain, blurred vision, and difficulties focusing, and physical effects, general discomfort, fatigue, boredom, drowsiness, headache, dizziness, concentration difficulties, and nausea. Participants answered reporting on a 6-point Likert scale the presence of symptoms, higher scores indicate more severe symptoms. The original version of the scale was validated by Ames^64^, reporting that the maximum irrelevant difference was set at 0.2.

### General screening, cognitive tests

A trained psychologist administered to the participants the Italian versions of several questionnaires. Questions about general health (e.g., presence of psychiatric, neurological, or oncological conditions; motor or visual problems) were collected to screen the presence of clinical conditions that may interfere with the study. Specific cognitive abilities were assessed as summarized in Table 1 Italian version of questionnaires were administered.

**Table 1:** Cognitive tests used within the study.

Variable	Test	Original scale citation	Italian scale used for the study	Validity/reliability of the instrument
Visual memory	Brief visual memory test revised (BVMT)	Benedict et al.^65^	Argento et al.^66^	Test–retest reliability r = from 0.60 for trial 1 to 0.84 for trial 3
Verbal fluency	Category fluency	Benton et al.^67^	Zarino et al.^68^	Test–retest reliability r > 0.75
Processing speed	Trail making test (TMT)—part B	Armitage^69^	Gaudino et al.^70^, Giovagnoli et al.^71^	Not reported
Working memory	Digit span backward	Weiss et al.^72^	Monaco et al.^73^	Test reliability = 0.89
Processing speed and visual perception	Symbol search (SS)	Weiss et al.^72^	Orsini et al.^74^	Test reliability = 0.88

### Data analysis

Descriptive analyses were performed on the collected data. Cognitive test scores were also evaluated by calculating the standardized z-score for the participants considering the normative data. Three trained observers evaluated the real version of VRAT (Real Action Test; RAT) performance (i.e., accomplishment and errors). Where there was a different evaluation between the observers, an agreement was found after discussion according to the NAT manual.

Pearson correlations were performed to compute the bivariate correlations between the key variables of the study, using jamovisoftware^75^.

In order to test our hypothesis, the models were analyzed by employing variance-based structural equation modeling (VB-SEM; known as partial least squares analysis), which was performed with the WARP PLS v.8.0 statistical software^76^. In VB-SEM, measurement error is explicitly modeled through the construction of latent factors, much like a covariance-based SEM analysis. VB-SEM, on the other hand, estimates models using ranked data, which is distribution-free, unlike covariance-based SEM. Model complexity, sample size, and deviations of the variable distributions from normality have less impact on model estimation. According to published criteria for VB-SEM models, VB-SEM analysis can evaluate the model at the measurement and structural levels. At the measurement level, VB-SEM establishes construct validity of the latent factors using the average variance extracted (AVE) and the composite reliability coefficients (ρ), which should exceed 0.50 and 0.70, respectively. AVEs for latent variables support discriminant validity if their square roots exceed the correlation coefficient with other latent variables. At the structural level, VB-SEM estimates the overall adequacy of the set of hypothesized relations among the model constructs using the goodness-of-fit (GoF) index given by the square root of the product of the AVE and average R^2^ for the model with values of 0.10, 0.25, and 0.36 correspond to small, medium, and large effect sizes for model fit, respectively^77^. The average path coefficient (APC) and average R^2^ (ARS) coefficients provide additional information about the model’s adequacy, both of which should be statistically significantly different from zero. Furthermore, the full collinearity variance inflation factor (AFVIF) is used to measure the level of multicollinearity, with values lower than 3.300 indicating that there are no issues with multicollinearity. Missing data were imputed using linear regression interpolation as recommended^78^. According to our hypothesis, we tested two moderation models, evaluating the role of sense of presence as a moderator in the relationship between (1) “Memory Moderation Model”: cognitive tests related to the memory (i.e., BVMT, Category fluency, and Digit span) domain and VRAT performance and (2) “Processing Speed Moderation Model”: cognitive tests related to processing speed (i.e., TMT, SS) and VRAT performance. We adopted an alpha level of 0.05, however, given the small sample size we also reported a marginal effect considered as < 0.10.

## Results

### Characteristics of the sample

On average, participants were 50.1 years old (SD = 2.2; range = 20–80). The sample comprised 54% of females. According to the Jack & Bondi guidelines^79^, only two participants showed a low cognitive impairment (for a complete characterization of participants, and the dataset of the study, see supplemental material Table S1 at the following link: http://osf.io/rcmyq).

### Cognitive test

Raw cognitive test scores, along with age and education-adjusted normative-based z-scores, are reported in Table S1 (http://osf.io/rcmyq). Scores on most tests of specific abilities fell within the average range, including tests of visual memory, verbal fluency, working memory, processing speed, and visual perception.

### Correlations

The correlation matrix (Table 2) showed a strong correlation between VRAT Scores with RAT scores. Specifically, the VR accomplishment shared 55.5% of the variance with Real scores (r = 0.745, *p* < 0.001), on the same page, VR error and Real error shared 78.5% of the variance (r = 0.89, *p* < 0.001). Alongside these results, the relation between cognitive tests and both versions of the NAT showed a coherent picture, in which high performances were strongly related to accomplishment in a positive direction and negatively with errors. Specifically, the accomplishment score in VR showed a pattern of strong correlation (r > 0.40) with all of the cognitive tests, while the errors score in VR showed a stronger correlation (r > 0.60) with symbol Search and TMT, and a lower correlation with memory tests (i.e., Digit span, Category fluency, BVMT). Considering the scores in real, the correlation matrix showed a similar pattern with VR scores, while a lower intensity for the relationship between accomplishment and cognitive tests than VR scores. The micro-errors in RAT correlated positively with errors in both conditions (i.e., VR and Real) and negatively with accomplishments in both conditions. Moreover, the micro-errors in RAT correlated with micro-errors in VRAT showing a Pearson index of 0.41. Concerning micro-errors in VRAT, the correlation matrix showed no significant relationships with the other parameters. Furthermore, age was significantly and positively correlated to errors in both conditions and inverse related to accuracy in the real one, however, did not show any other correlation besides an inverse correlation with BVMT.

**Table 2:** Correlation matrix between key variables of the study.

	Age	BVMT	Category fluency	Digit span	Symbol search	TMT	Acc VR	Error VR	Acc Real	Error real	Micro-error real	Micro-error VR
Age	–											
BVMT	− 0.653**	–										
Category fluency	− 0.075	0.392	–									
Digit span	− 0.144	0.258	0.151	–								
Symbol search	− 0.214	0.368	0.492	0.410	–							
TMT	− 0.004	0.425	0.450	0.166	0.524	–						
Acc VR	− 0.489	0.429	0.560*	0.518*	0.565*	0.466	–					
Error VR	0.536*	− 0.380	− 0.430	− 0.189	− 0.624**	− 0.698**	− 0.685**	–				
Acc Real	− 0.552*	0.305	0.450	0.297	0.563*	0.142	0.745***	− 0.663**	–			
Error real	0.586*	− 0.548*	− 0.425	− 0.300	− 0.713**	− 0.619*	− 0.581*	0.886***	− 0.712**	–		
Micro-error real	0.277	− 0.109	− 0.112	0.210	-0.333	0.140	− 0.217	0.479	− 0.446	0.384	–	
Micro-error VR	0.247	− 0.017	0.126	0.324	0.239	0.142	0.150	0.105	0.180	0.016	0.416	–

*Note.* BVMT brief visual memory test, *TMT* trial making test; *Acc VR* accomplishment in virtual reality, *Error VR* error in virtual reality, *Acc Real* accomplishment in real condition, *Error Real* error in real condition.

*p < .05, **p < .01, ***p < .001.

### Moderation models

According to our hypothesis, the moderation models exhibited acceptable fit: “Memory Moderation Model” (GoF = 0.868; APC = 0.277, *p* = 0.05; ARS = 0.783, *p* < 0.001; AVIF = 1.579) and “Processing Speed Moderation Model” (GoF = 0.786; APC = 0.422, *p* = 0.01; ARS = 0.649, *p* < 0.001; AVIF = 3.483).

The “Memory Moderation Model” showed that the effects of BVMT on the VRAT score were marginally moderated (*p* = 0.07) by the sense of presence, positively with accomplishments and negatively with errors. All the paths between cognitive test and VRAT performance were significant or marginally significant and coherent in the direction.

**Figure 1:**
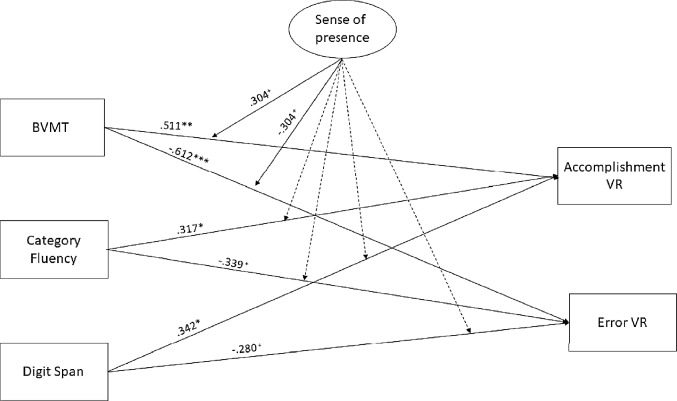
Estimates of the structural equation model involving memory domain. *Note* *BVMT* brief visual memory test, *Accomplishment VR* = accomplishment in virtual reality, *Error VR* error in virtual reality. Dashed lines refer to nonsignificant path estimates. ^+^*p* < 0.10, *p < 0.05, **p < 0.01, ***p < 0.001.

**Figure 2:**
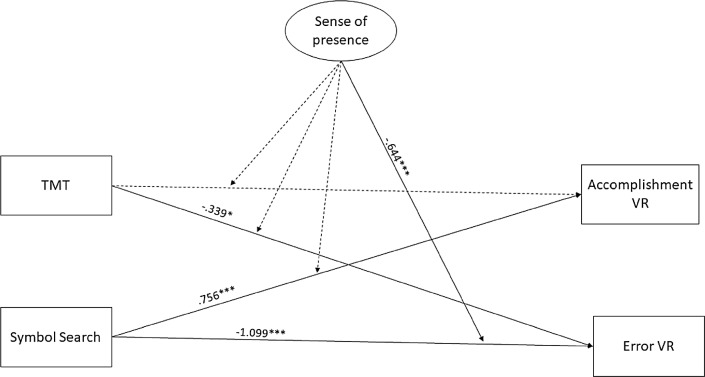
Estimates of the structural equation model involving processing speed domain. *Note* *TMT* trial making test, *Accomplishment VR* accomplishment in virtual reality, *Error VR* error in virtual reality. Dashed lines refer to nonsignificant path estimates. *p < 0.05, **p < 0.01, ***p < 0.001.

The “Processing Speed Moderation Model” showed that the statistically significant effect of the Symbol search test on VRAT errors was significantly and negatively moderated by the sense of presence. Concerning the direct effects of the cognitive variables on the VRAT performances, the Symbol Search was significantly and positively related with accomplishments, and negatively with errors, while TMT showed a similar pathway, significant only with the errors performance. The path coefficients and the *p* values of the models are reported in Figs. 1 and 2.

### Cybersickness

Before starting the procedure none of the participants reported any motion sickness symptoms related to any visual devices (e.g. TV, video game). Cybersickness was evaluated post-test with VRSQ (scale range 0–6), and the results showed no significant level of cybersickness symptoms of all the participants (Physical symptoms: M = 0.2; SD = 0.6; Eye symptoms: M = 0.5; SD = 0.8).

## Discussion

The main purposes of the study were: first, to evaluate the validity of the Virtual immersive version of the NAT; second, to evaluate the effect of cognitive tests in predicting the VRAT performances, considering also the role of sense of presence as moderator.

In the scientific literature, other scholars digitalized the NAT using not immersive technology with good results^43^. Following this mainstream, Chirico et al.^11^ developed a virtual immersive version of NAT (VRAT) implemented through Head-Mounted display devices, demonstrating the feasibility of the VRAT in a single case study. The results of the study supported the idea of implementing the VRAT even in people with minimal computer experience or with no prior VR exposure, without any significant risk. Furthermore, from a descriptive point of view, the results of both conditions, virtual and real, suggested similar outcomes^11^. In line with these suggestions, the present study evaluated the concurrent validity of the VRAT and NAT in a cross-over design study, relying on a sample of 16 participants evaluated on memory and processing speed domains. Correlation analyses showed significant relations between VRAT scores (i.e., accomplishments and errors) and (1) RAT scores (i.e., accomplishments and errors), and (2) tests of cognitive abilities. These results suggested the potential validity of the immersive VRAT for function assessment, as the correlations of the scores (i.e., accomplishment and errors) between the different conditions (i.e., VR and real) showed a high Pearson index (> 0.70). This result is in line with previous evidence supporting the validity of the digitalized automated version of the NAT, where the correlation was similar, but lower (r = 0.47)^43^ probably due to the non-immersive digitalization (computer based) of the test In line with our hypothesis, the results showed that the participants made similar scores in the virtual and real versions of the task, and concurrent validity with neuropsychological measures. This finding showed a good convergent validity with high significant correlations (> 0.7), consistent with what has been observed in previous validation studies of where virtual reality has been evaluated in relation to neurocognitive assessments (see meta-analysis of Neguț et al. 2015)^80^. Furthermore, high correlation between virtual and real version of the same task (i.e. Naturalistic Action Test) are in line with previous studies, showing higher correlation among the VR and Real version of the instrument (r = 0.95)^81^. To speculate, a possible reason could be retrieved in the development of the scenario. The virtual environment was designed to mimic the real environment, resulting in the objects and their configuration being identical to those found in the real version of the task. As well as the procedure was exactly the same in both conditions.

Regarding the micro-errors, the correlation matrix showed different patterns in the two conditions. Specifically, the micro-errors in RAT showed a coherent pattern with the other scores, although the sample size has influenced the *p* values of the correlations. Indeed, the micro-errors correlated positively with errors and negatively with accomplishments in both real and virtual conditions. Moreover, the micro-errors in RAT correlated with micro-errors in VRAT. Concerning micro-errors in VRAT, the null relationships reported in the correlation matrix could be attributed to the difficulty of the software detecting micro-errors in a virtual environment. Indeed, the automated scoring was inaccurate in tracking subtle functional difficulties. Automatic scoring was shown to be overly sensitive to the hand movements of the participants, leading to inaccurate results. The progress of technology may improve the accuracy in detecting micro-errors, the authors of the present study are involved in a new protocol in order to fix this issue. Despite the difficulties mentioned above, the software was accurate in tracking errors and accomplishments, showing promising results.

Correlation analyses were performed between RAT scores and cognitive abilities, besides the relation between error and digit span, all variables resulted in statistically significant or marginally significant correlation. Although not all correlations were significant, given the Pearson indices, it is reasonable to think that the small sample size could have influenced these results. In any case, despite the small sample size, these results are in line with the literature^40,41,43,82–84^, highlighting the preliminary validity of the RAT test.

As expected, also the correlation matrix between VRAT measures and tests of cognitive abilities followed the same pattern of the RAT’s correlation with cognitive tests, suggesting the accuracy of the automated score in detecting the accomplishments and errors during the task. These results are in line with a previous study^43^ related to the validation of a virtual non-immersive version of NAT and other VR neuropsychological measures^85,86^.

Taken together, these results provided preliminary validity data for the VRAT and suggest the utility of the VRAT as an objective and efficient measure of functional difficulties. Virtual immersive technology allows to develop and implements immersive virtual scenarios in which, unlike non-immersive technology, the users could be able to experience the same feelings as the real scenarios. The role of VR experience is still debated in the scientific literature^44^. Specifically, previous studies investigated the role of the sense of presence experienced by participants during the VR tasks^49–51,87^, suggesting that it is often complicated to find a direct effect on scoring^46^.For this reason, it becomes important to evaluate the sense of presence experienced by participants testing its role in moderating the effect of cognitive tests on VR tasks, when this can be applied.

To the best of our knowledge, this is the first study that evaluates the role of VR presence in moderating the relationship between cognitive abilities and the score of performance-based tests in virtual immersive environments. Our results showed that the sense of presence experienced by the participants during the task moderated the effects of BVMT on VRAT scores and the relationship between symbol search and errors. Although the relationships between sense of presence and performance were not found in previous studies^51,87^ or were found weak associations^49,88^, our findings highlighted that the VR presence may have a moderation effect in these contexts. On the same page, the highest direct effects in the two models showed a pattern that emphasizes the role of visual components in our VR task. As a matter of fact, our results highlight as both cognitive tests related to the visuospatial domain (i.e., visuospatial memory; BVMT, and visuospatial attention; symbol search) had the highest effects on VRAT performances and were the only to being moderated.

Maneuverer et al.^59^ tested the effect of the sense of presence in predicting spatial cognition performance in an immersive virtual test (Rod-and-Frame Test; RFT). The authors found a positive effect of the sense of presence and other variables (e.g., cybersickness, game experience) on the participant's performance. Moreover, the scholars tested a mediation model without any significant results. However, they highlighted the role of human factors (e.g., sense of presence, cybersickness, game experience) in the spatial immersive virtual tasks. Following this claim, our study provides an empirical contribution to the role of sense of presence as a moderator, considering the validation of the VRAT test in performing daily living activities with no spatial performances involved. Our results showed that the sense of presence was not a predictor of VRAT performances. However, correlation analysis showed a null effect in the relation between sense of presence and cognitive domain and VRAT performances. To speculate, these results can be explained by the fact that given the different mechanisms involved in the NAT, where spatial performances are not involved to complete the task, the sense of presence becomes a crucial booster of expressing cognitive abilities (i.e., daily living activities) within the VE. Moreover, participants reported no symptoms of cyber sickness in VRAT, suggesting the feasibility of the VR devices for all of them. The present study posed some important questions related to the complex interaction between sense of presence and performance in virtual reality tasks. Although the present study reported preliminary data, our results represented a first step in understanding the process behind the individual variations of sense of presence without any manipulation of the immersive levels provided by the technological factors.

The present study is not without limitations. First, our findings should be interpreted with caution due to the low sample size, which may limit the generalizability of the results. The sample collection was carried out just before the COVID-19 pandemic, so the collection data was unexpectedly and unpredictably stopped because of the worldwide COVID-19 lockdown, reducing the number of participants involved in the present study. Moreover, when the restrictive measures to prevent the COVID-19 spread were ease, we tried to recruit new participants in increasing the sample size. Unfortunately, many people were still concerned about their health and safety and rejected to participate in our research. However, we used PLS structural equation modeling, especially considering the low number of participants. The use of the PLS structural equation model provides features specifically aimed at increasing accuracy and statistical power through resampling. In our study, we used a “stable” resampling method that tends to generate low standard errors, with small samples and medium-to-high effect sizes, particularly we adopted the “Stable3” method^89^. Future studies should investigate the moderating effect of a sense of presence with a larger sample size to confirm our results. Second, the present study was not aimed at evaluating the VRAT as a discriminating neuropsychological disorder. Our aimed was to evaluate the validity of the VRAT on a healthy sample considering the role of sense of presence and being aware of the variability of this variable in the clinical population due to their symptoms. Future studies could test the discriminant validity of VRAT considering the clinical population (e.g., people with cognitive impairment).Third, the manipulation of the objects in the virtual environment was facilitated by a visual feedback in order to inform the users that the object is selected and interactable with the controller. This feedback is not available in real world conditions in which the NAT may traditionally be applied. In order to enhance the natural and intuitive interaction with virtual environments, upcoming research could implement innovative devices like the Motion Capture already integrated in newest devices. At the same time, the interaction in the virtual world, is not as simple as in the real world, given the controller needed to interact with objects. For these reasons, other studies should compare visual feedback (with no feedback) and the use of controllers versus motion capture.

Future studies should also take into account the interaction between human factors and VR systems, focusing on the relationships between several human factors (e.g., cybersickness, gender, VR experience) and different levels of immersive technology, maybe varying the immersive levels of the virtual environment. Although several studies mentioned above have evaluated the direct effect of human factors on performances in VR tasks, future researchers should consider these factors as a moderator component in the resolution of the task in a virtual environment (e.g., performance-based test in VR). Although the present study showed promising results, given the small sample of participants, broader studies are needed to confirm these findings.

### Supplementary Information

Supplementary Table S1.

## Data Availability

Data are available at the following link: http://osf.io/rcmyq.
